# Wildlife Crime: Issues and Promising Solutions

**DOI:** 10.3390/ani12141736

**Published:** 2022-07-06

**Authors:** Stephen F. Pires, George Olah

**Affiliations:** 1 Department of Criminology & Criminal Justice, Florida International University, Miami, FL 33199, USA; 2 Fenner School of Environment & Society, The Australian National University, Canberra, ACT 2600, Australia; george.olah@anu.edu.au

## 1. Introduction

The poaching of wildlife for profit, pleasure, subsistence, or as a result of human–animal conflict has decimated wildlife populations—particularly those of at-risk species. Given the ease of poaching wildlife throughout the world and the significant demand for specific species and their derivatives, wildlife trafficking is among the most profitable illicit transnational industries [1]. Seizure and government reports have shown a steady increase in wildlife trafficking, and many of the most endangered species continue to experience population declines. Despite technological advancements to detect and prevent poaching and trafficking, the current approaches to curb the trade have had limited success or are not working. This Special Issue on “Wildlife Crime: Issues and Promising Solutions” had a broad objective of soliciting empirical research on the nature of the illegal wildlife trade; novel solutions to track, prove, and prevent wildlife crime; and evaluative research on enacted legislation. It offers six new research studies thanks to 34 authors from 14 institutions based in North America, Europe, and Australia. Altogether, this volume of work is an effort to contribute to our understanding of how the illicit trade operates and provide viable solutions to mitigate threats to protected wildlife.

## 2. Genetics in Wildlife Forensics

Genetic methods have been widely used in human forensic investigations, but these techniques are rarely applied to wildlife forensics, with some exceptions of illegally traded wildlife products of high financial value (e.g., ivory and domestic species). Two studies in this SI show examples of how modern genetics can be applied to cases of wildlife forensics. Rodionov et al. [2] present a case study where western tur *Capra caucasica*, an endangered goat antelope native to the Caucasus Mountains, was poached. In February 2020, outside of the legal hunting season, remnants of a goat/tur were found in the Caucasus Mountains. At the same time, the local police confiscated meat and bones from a suspected poacher, who claimed that these belonged to his flock containing domestic goat *Capra hircus* and hybridized individuals with western tur. The authors applied genome-wide SNP genotyping using DNA chips for species assignment, which clearly identified the remnants and confiscated materials as wild western tur. Furthermore, they used 14 STR (microsatellite) genetic markers and concluded that both the remnants at the crime scene and the confiscated evidence from the suspect belonged to a single male western tur. This study, using DNA chips for the first time in wildlife forensics of ungulates, is a good demonstration of the advantages of using multiple genetic techniques in wildlife forensic cases.

In another paper, Zenke et al. [3] developed and forensically validated a simple, fast, and cost-effective genetic assay to identify the species and sex of abundant European game species, including red deer *Cervus elaphus*, roe deer *Capreolus capreolus*, and fallow deer *Dama dama*. There has been a considerable amount of abuse inflicted on these antlered game species, so developing a new technique for the identification of their unidentifiable remnants was essential. Hunting laws not only determine illegal time periods for harvest, but often specify the sex of the animal that can be legally culled during a certain period, which also influences the shooting prices. Hence, the hunted species and their sex are often falsely reported. The authors developed species-specific primers on the Cytb gene, resulting in different fragment lengths for each of the three species. For sexing, two conserved regions of the Y-chromosome were used simultaneously, amplifying different fragments for males and females (and also different by species), providing an internal control. Techniques such as this can provide early and cost-effective screening of illegal activities related to game and trophy hunting.

## 3. Conservation Crime Science

Two articles within this Special Issue focus on wildlife poaching risk from a conservation crime science approach—a branch of criminology that studies crime events and their contexts in order to prevent conservation problems [1]. In the first article, Petrossian and associates [4] set out to study the impact of potential illegal longline fishing vessels on the outcome of albatross endangerment at high seas. Albatrosses (Diomedeidae) are one of the most threatened bird species in the world due, in great part, to the risk that longline fishing vessels present to such seabirds in the form of hooking albatrosses or entangling them in gillnets by accident. However, not all longline fishing vessels are equal in relation to albatross risk. Illegal fishing vessels are less likely to use bycatch mitigation measures that would reduce the likelihood that albatrosses are killed. To empirically examine whether such vessels are spatially associated with an elevated average IUCN risk score of albatrosses, a multivariate spatial econometric model was created that considered the presence or absence of potentially illegal longliners, legal longliners, along with a cumulative weighted score of the probability of finding commercially sought-out fish. The results suggest that both commercially sought-out fish and the presence of potential illegal longliners are spatially clustered in similar parts of the world. Further, the average risk to albatrosses was significantly higher in areas where potential illegal longliners were present, while controlling for the presence of legal longliners. This suggests that potential illegal longliners pose a greater risk to albatross endangerment than legal longliners, a finding that had not been empirically examined to date. The implications of these findings point to spatially targeted interventions, where the distribution of the greatest number of endangered albatross species intersects with potential illegal longliner presence.

In the second article, Viollaz and colleagues [5] interviewed stakeholders in a small village in a South African province to uncover the reasons why retaliatory leopard killings occur in rural farming contexts. Human–animal conflict is a common problem throughout the world (e.g., predation on livestock) that often leads to retaliatory killings affecting a variety of species. Through 16 interviews with various stakeholders, several themes emerged from discussions with both government actors and farmers. From the perspective of the farmers, their distrust of the government, their solutions, and their lack of willpower to help farmers were impediments to joint problem solving. Surprisingly, “there was a significant amount of unacknowledged agreement on what solutions did or did not work” between both parties, but because both parties distrusted each other, effective solutions were difficult to implement in the field [5] (p. 7). By asking both farmers and government actors what solutions appeared to be effective (and ineffective), potential solutions—guided by situational crime prevention—were highlighted by Viollaz and associates. One such solution considered “giving farmers financial incentives to conserve leopards” by setting “aside land for conservation and implement predation prevention techniques, giving farmers an incentive to tolerate some livestock predation” [5] (p. 11). Solutions such as this, among others that were recommended, highlight the importance of positive incentives for behavior change that are less frequently implemented in the field of conservation. By focusing on a case study of leopard retaliatory killings, potential solutions to and insights into this problem can be applied elsewhere to a variety of other species.

## 4. Law and Wildlife Trade

Wildlife market surveys are an important tool in identifying and tabulating the number of specimens by species that are routinely trapped and traded from the near or greater regional area. However, identifying what species have been trapped and traded that are compliant with domestic laws and regulations is less clear due to a variety of regulations and unknown provenances of species. In their article, Nijman and colleagues [6] suggest that several factors need to be considered with regard to the legality of the wildlife trade that is not limited exclusively to protected species legislation or the CITES listing of exported species. Researchers should consider a broad array of potential violations that include “(1) protected species, (2) harvest quota, (3) welfare, (4) transport restrictions, and (5) importation” [6] (p. 4). Using five case studies of various bird species in Indonesia, the investigation of wildlife markets—both on-site and online—made it possible to identify violations of domestic laws along with the magnitude of the trade. The results found that each of the five categories were violated at least once, with *transport restrictions* being the most violated regulation in four of the five case studies. This study demonstrates the importance of wildlife market surveys to understand the nature of the wildlife trade, both legal and illegal, as a valuable methodological tool that is both reliable and valid.

Parrots are the most traded group of birds, whose wild populations have been suffering great losses [7]. However, the laws and regulations regarding the legal and illegal aspects of this trade are often confusing and contradicting between national and international levels. Romero-Vidal et al. [8] made the first attempt to disentangle such regulations regarding the domestic and international wildlife trade in Neotropical countries, specifically focusing on the trade and possession of parrots. They found that among 46 Neotropical countries/territories with native parrot populations, only Suriname and Guyana currently allow the capture, trade, and possession of these parrots, while Suriname, Guyana, and Peru also allow legal international trade. They identified many cases of transboundary smuggling of parrots. The authors conclude that “one of the main problems in regulating international wildlife trade is the implementation of the main regulatory mechanism (CITES), as each signatory country is a sovereign state and is responsible for implementing law enforcement measures within its territory”. In addition, they highlight that “a notable point of confusion is that about half of the countries maintained legal exports years or even decades after prohibiting the capture of parrots for the domestic demand”. Such discrepancies and the complexity of laws regulating wildlife trade activities can confuse authorities and customers, increasing the conservation problems posed to wildlife. In light of this study, better collaboration between Neotropical countries, new tools to efficiently investigate wildlife crime, better awareness raising, and stronger enforcement of the current regulations are needed to halt parrot and other wildlife trade in the region.

It is our hope that this Special Issue on wildlife crime spurs more research in the field to better understand the nature of the illegal wildlife trade and prevent it.
